# Nanotechnology-Based Drug Delivery Systems in the Transdermal Treatment of Melanoma

**DOI:** 10.34172/apb.2023.070

**Published:** 2023-01-23

**Authors:** Zahra Saeidi, Rashin Giti, Mehdi Rostami, Farhad Mohammadi

**Affiliations:** ^1^Department of Pharmaceutics, School of Pharmacy, Shahid Sadoughi University of Medical Sciences, Yazd, Iran.; ^2^Department of Prosthodontics, School of Dentistry, Shiraz University of Medical Sciences, Shiraz, Iran.

**Keywords:** Cancer, Liposomes, Malignant, Melanoma

## Abstract

The incidence rate of melanoma is dramatically increasing worldwide, raising it to the fifth most common cancer in men and the sixth in women currently. Resistance generally occurs to the agents used in chemotherapy; besides their high toxicity destroys the normal cells. This study reviewed a detailed summary of the structure, advantages, and disadvantages of nanotechnology-based drug delivery systems in the treatment of melanoma, as well as some nanocarrier applications in animal models or clinical studies. Respective databases were searched for the target keywords and 93 articles were reviewed and discussed. A close study of the liposomes, niosomes, transferosomes, ethosomes, transethosomes, cubosomes, dendrimers, cyclodextrins, solid lipid nanoparticles, and carbon nanotubes (CNTs) was conducted. It was found that these nanocarriers could inhibit metastasis and migration of melanoma cells and decrease cell viability. Conclusively, some nanocarriers like liposomes, niosomes, and transferosomes have been discussed as superior to conventional therapies for melanoma treatment.

## Introduction

 The malignant disease of melanoma has increasingly occurred for decades and is predicted to continue rising. It is 1.5 times more prevalent in men than women and notably age-dependent, occurring more frequently in women under 40 and about 3 times more common in men than women under 75 years of age.^1,2^ Unlike many solid cancers, melanoma generally affects young and middle-aged individuals (median age = 57).^3^ Despite accounting for only 4% of skin neoplasms, melanoma is the most aggressive cancer that constitutes more than 80% of skin cancer-related mortalities.^4,5^ The United States report it as the fifth and sixth most common cancer in men and women, respectively; being the 12^th^ and 11^th^ in the United Kingdom. Its incidence rate rose from 6.8 to 20.1 per 100 000 persons from 1973 to 2007 in the United States. In 2011, it was seen in 25.4 and 15.7 per 100 000 men and women, respectively.^1,6,7^

 The earliest pathogenic event that initiates carcinogenesis in melanoma is the conversion of melanin from antioxidant to pro-oxidant in melanocytes due to some factors like UV radiation. This conversion increases the levels of intracellular oxygen radicals and consequently damages the DNA molecules in the melanocyte. These mutations activate various cell signaling pathways and result in uncontrolled proliferation and differentiation of specific cell types.^8^ The mitogen-activated protein kinase is the most important signaling pathway in the proliferation and apoptosis of cells in melanoma. It is wrongly activated and inhibits apoptosis due to different abnormalities like B-Raf proto-oncogene (BRAF) and neuroblastoma-RAS mutations. They are seen in 15% to 30% of the cases, about 80% of which relate to the 12, 13, and 61 codons. Mutation in the tumor suppressor gene of phosphatase and tensin homolog is seen in half of the melanoma cell lines. It rapidly decreases the signaling activity of phosphatidylinositol-3-kinase (which promotes cell proliferation). Mutation activates phosphatidylinositol-3-kinase signaling, produces anti-apoptotic proteins such as B-cell lymphoma, and reduces apoptotic proteins such as P14 and P16 (Table 1).^3,9^

**Table 1:** Food and Drug Association-approved drugs for treatment of melanoma

**Drug**	**Brand name**	**Target molecule**	**First FDA approval**
Aldesleukin	Proleukin	Interleukin 2 receptor	January 1998
Binimetinib	Mektovi	Mitogen-activated extracellular signal regulated kinase 1 (MEK1) and MEK2	June 2018
Encorafenib	Braftovi	BRAF kinase	September 2020
Cobimetinib	Cotellic	MEK1 and MEK2	August 2015
Dabrafenib	Tafinlar	BRAF kinase	May 2013
Dacarbazine		DNA/RNA	November 1975
Talimogene	Imlygic	Unknown	October 2015
Recombinant interferon alfa-2b	Intron A	Interferon α	June 1986
Trametinib	Mekinist	MEK	May 2013
Ipilimumab	Yervoy	CTLA4	March 2011
Vemurafenib	Zelboraf	BRAF kinase	August 2011
Pembrolizumab	Keytruda	PD-1	September 2014
Nivolumab	Opdivo	PD-1	December 2014
Tebentafusp	Kimmtrak	Gp100	January 2022

 Melanoma is divided into cutaneous and non-cutaneous types, the latter of which constitutes only 5% of all cases but is more aggressive than the cutaneous type (25% vs. 81% survival rate). Cutaneous melanoma occurs in four main types of superficial spreading melanoma, nodular, lentigo maligna, and acral lentiginous. Non-cutaneous melanoma is seen in two main types: ocular (73%) and mucosal (including gastrointestinal, biliary, and anorectal). Ocular melanoma mostly appears in the iris, uveal tract of the choroid, and ciliary body. Mucosal melanoma is most frequently detected in the genital tract.^6,10^ The American Joint Committee on Cancer defines four stages for melanoma based on the assessment of the primary tumor, regional lymph nodes, and distant metastatic sites. The highly curable stage one is considered as a localized form of melanoma, stage two is also a localized form but with a high risk of recurrence, in stage three, melanoma spreads to the regional lymphs, and in stage four, melanoma spreads beyond the regional lymphs.^11^

 Although localized melanoma can be treated in its early stages, the metastatic type is poorly diagnosed and does not fully respond to conventional therapies such as chemotherapy, radiotherapy, and surgery. Despite the potency of chemotherapy in destroying cancer cells, tumor stem cells can survive and develop drug-resistant melanoma. Dacarbazine and temozolomide are two common alkylating agents in chemotherapy, but they have limited benefits for metastatic melanoma. Complete response with dacarbazine is only achieved in 5% of patients and overall survival has never been reported in clinical trials.^5,12^ In the early stages of melanoma, surgery is the best intervention therapy in which the lesions will be removed with a certain safety margin. However, it may commonly fail due to tumor relapse and not be appropriate in some types like oral and nasopharyngeal melanoma.^13,14^ Meanwhile, radiation therapy is done via high-energy radiation to cause cell death.^15^

 Immunotherapy agents improve the host’s immune response in the clearance of cancer cells. The most commonly-used agents are Interleukin 2 which stimulates T cell proliferation and Interferon alpha; the former was approved by the Food and Drug Association in 1998 for the treatment of metastatic melanoma. However, it acts poorly in low doses and subcutaneous use and is only efficient in less than 20% of cases.^5,13^ Two other agents are checkpoint inhibitors like CTLA-4 and PD-1 inhibitors.^15^ Hyper-activated pathways provide a therapeutic way which is targeted therapy. BRAF inhibitors can be used in targeted therapy, although they have not shown any promising results and showed little efficacy per se or in combination with chemotherapeutic agents. A common strategy against melanoma is combination therapy, which increases efficacy and side effects simultaneously; as in combination of dacarbazine with other cytotoxic drugs such as cisplatin and carmustine.^5,12^ The combination of dacarbazine, carmustine, cisplatin, and tamoxifen and the combination of cisplatin, vinblastine, and dacarbazine is called Dartmouth and CVD regimen, respectively.^13^

 Active targeting refers to the interaction between functionalized drug-loaded nanoparticles (with targeting ligands including antibodies, peptides, nucleic acid, and small molecules) and the receptors or molecules highly expressed in targeted tumors. Some of the highly specific biomarkers of melanoma are intracytoplasmic proteins with a low expression on the surface; thus, receptors like transferrin and folic acid that overexpress on the surface of solid tumors like melanoma can be used for targeted drug delivery. Receptors like melanocortin receptor-1, fibroblast growth factor receptor, laminin receptor, somatostatin receptor, and sigma receptor show a high expression in melanoma and a lower expression in normal cells. By using both the therapeutic and diagnostic capability of nanoparticles, nanotheranostics have become a new strategy, especially for imaging the localization of early metastatic melanoma, detecting the nanoparticles’ distribution, and delivering the therapeutic agent to melanoma cells. For surface modifications of nanotheranostics, chemical functionalization and biofunctionalization can be used. Chemical functionalization is the conjugation of chemical ligands, therapeutic drugs, and imaging agents to the nanomaterial. Some chemical approaches like amid coupling, thiol coupling, and PEGylation can be utilized. Biofunctionalization is the use of some natural bioresources like bacteria and plants.^16,17^

 This review aimed to summarize and discuss nanotechnology-based drug delivery systems with a detailed summary of their structures, advantages and disadvantages, and some nanocarrier applications in animal models or clinical studies.

## Search strategies

 PubMed, Google Scholar, Scopus, and Elsevier databases were searched for the keywords nanotechnology systems, melanoma, transdermal therapy, cancer, nanocarriers, liposomes, niosomes, transferosomes, ethosomes, transethosomes, cubosomes, dendrimer, and cyclodextrins. Out of 165 review and research articles, those published before 2000 (n = 42) and with irrelevant titles and abstracts (n = 30) were excluded; 93 articles were reviewed and discussed.

## Nanotechnology-based drug delivery systems

 The use of nanotechnology for drug delivery and cancer imaging can improve efficacy and reduce toxicity. Nanostructure protects the administered drug by prolonging blood circulation and, thereby, improves the drug accumulation in the pathological tissues.^18,19^ The most important nanotechnology-based drug delivery systems as transdermal carriers are vesicular systems such as liposomes, niosomes, transferosomes, and particular systems like polymeric nanoparticles dendrimers, magnetic nanoparticles, carbon nanoparticles, gold nanoparticles, and silica nanoparticles (Tables 2 and 3).^20^

**Table 2:** Main studies and main outcomes of nanotechnology-based drug delivery systems

**Nanotechnology-based drug delivery systems**	**Anticarcinogenic agents**	**Target molecule**	**Functionalization**	**Characterization**	**Cell culture outcomes**	**Animal model outcomes**	**References**
Liposomes	Vemurafenib	V600E mutated BRAF protein	Peptide TD modification	Liposomes were biocompatible, highly-stable with high loading efficacy of 98.92% The size of Vem-Td-lip was 106 nm and zeta potential -4.75 mV	Low cellular toxicity, and selective inhibition of A375 cell Td modification could enhance the permeation of vemurafenib	Safety studies showed oral and intravenous administration of Vem-TD-Lip in comparison with local administration significantly damaged the liver, kidney, and lung The tumor size, relative volume, and weight after two weeks administration of Vem-TD-Lip were significantly lower than PBS	^21^
Liposomes	PTX Hydroxychloroquine	Microtubules CXCR4/CXCL12 axis	R8-dGR peptide modification	Particle size 100 nm, polydispersity index less than 0.3, EE > 85%, release rate 30 and 60% over 48 h for hydroxychloroquine and PTX		The relative rates of wound healing were 4.82 ± 3.06% and 12.59 ± 4.15% after 24 h and 48 h, respectively. Liposomes had inhibitory effect on migration, and reduced the lung metastasis in-vivo	^22^
Niosomes	Artemether	Binds to heme molecule in cancer cells		The size of formulations was between 116-493 nm and EE = 50-82%. EE was higher in formulations with span 60 and 80 than tween as surfactants and EE in formulations with mixed tween and span was even higher.	The niosomal formulation in comparison with artemether reduced the tumor volume and increased the necrosis.	The inhibitory effect was improved by increasing the artemether concentration and a significant decrease in tumor volume was seen by day 6 in noisome-treated group compared with the free drug treated group	^23^
Transferosomes	PTX	Microtubules	Cell-penetrating-peptide-modification	PTX- CTs size was 75 nm with the charge + 21mV. The drug-loading capacity and entrapment efficiency of PTX in the PTX-CTs were about 3.2% and 99%, respectively.	Tumor penetration and *in vitro* anticarcinogenic effect of PTX-CTs showed that the PTX-CTs had higher cytotoxic effect on the B16F10 cells compared with other PTX formulations.	The Cou6 permeation through the skin area was about 11.4% in the Cou6-loaded transferosome within 12 h versus 4.8% of the Cou6-loaded liposome.	^24^
Ethosomes	Mitoxantrone	DNA		The size and zeta potential of the ethosomes were 78 nm and -55 mV, respectively.	The cell uptake of mitoxantrone from the ethosome gel was high.	In-vitro permeability across rat skin and anti-melanoma effect of mitoxantrone ethosomal gel was higher in comparison with the mitoxantrone solution. The tumor inhibitory rate of the mitoxantrone ethosome gel was 68.44%	^25^
Cubosomes	Doxorubicin	DNA topoisomerase2	Radiolabeled with 177Lu through conjugation with DOTAGA-OA	Blank cubosome, DOTAGA-OA-loaded cubosomes, DOTAGA-OA and doxorubicin had the size of 181, 153, and 160 nm, respectively, and zeta potential -27, -11, and -19 mV.	The combination of doxorubicin and B-emitting radionuclide in the form of cubosomes can improve cytotoxicity. The multifunctional cubosomes lead to the application of a small dose of the cytotoxic drugs and reduced their side effects.		^26^
Dendrimers	Re188	DNA	Conjugation with chelator agent Suc-HYNIC (Succinimidyl 6-hydrazinopyridine-3-carboxylic acid hydrochloride)		Aberrant metaphase was 29.5 % in cells treated with 15 μCi (0.555 MBq) of 188Re-dendrimer for 24 h compared with the control cell, which was about 7%.	Biodistribution assay exhibited high liver uptake (30 %) and renal clearance in melanoma bearing mice.	^27^
Cyclodextrins	Harman	Sphingosine kinase-1		EE was 88.97% SEM micrographs of βCD-HAR system showed homogenous rectangular block structures. FTIR showed that HAR had the intermolecular hydrogen bond with βCD cavity.	βCD-HAR promoted higher toxicity than HAR. HAR and βCD-HAR decreased the migration rate by 32.23% and 46.37%, respectively. Caspase-3 activity was higher in cells treated with βCD-HAR compared with HAR treated cells.		^28^
Solid lipid nanoparticles	PTX	Microtubules	Tyr-3-octreotide (PSM)	The mean diameter was 119.4-160.9 nm and zeta potential between -25.4 and -34.1 mV.	The necrosis and apoptosis rate were higher in the PSM treated group compared with dacarbazine treated group.	PSM showed the least lung metastasis in mice models. The final size of the tumor in the PSM-treated group was much less that of DTIC treated group.	^29^

**Table 3:** Clinical studies and main outcomes of nanotechnology-based drug delivery systems

**Nanotechnology-based drug delivery systems**	**Anticarcinogenic agents**	**Target molecules**	**Functionalization**	**Characterization**	**Cell culture outcomes**	**Clinical outcomes**	**Reference**
Liposome	Arachidonyl trifluoromethyl ketone 25	Cytosolic phospholipase A2		EE was 62%, 70% of arachidonyl trimethyl ketone was released after 48-72 h. The size was 67.58 with the zeta potential -0.35.	Liposomes killed cancer cells more effectively than normal human cells and were about two times less toxic to the normal control cells in comparison with the melanoma cell lines.	Nano arachidonyl trimethyl ketone at 30-40 mg/kg led to significantly reduce the tumor volume by 58% and 55%.	^30^

###  Liposomes

**Figure 1:**
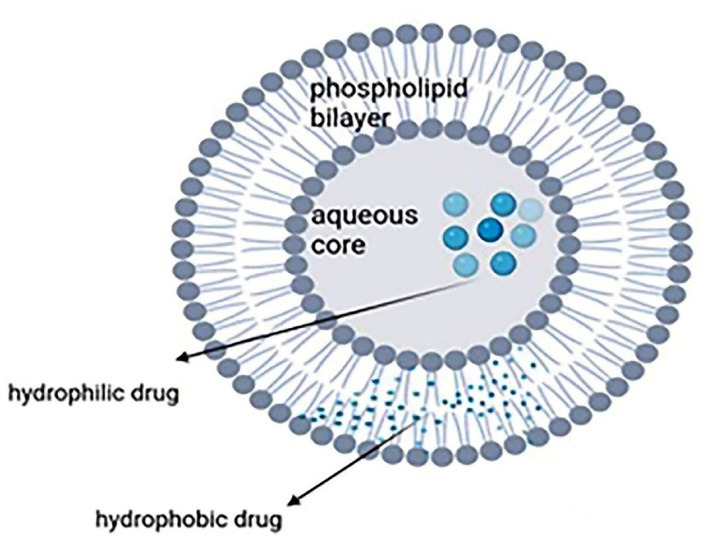
Structure of liposomes encapsulated with hydrophobic and hydrophilic drugs

 Liposomes are widely used in pharmaceutics because of their flexibility, efficacy, and various application routes like topical, intravenous, and oral. They are spherical microscopic nanostructures with an aqueous core surrounded by one or more phospholipid bilayers and a size from 10 nm to several micrometers depending on the construction technique. They can accommodate both hydrophobic and hydrophilic drugs because two internal and external phases are required to form a bilayer phospholipid surrounded by an aqueous component (Figure 1). Liposomes can be made from both natural (like lecithin and sphingomyelins) and synthetic lipids (like dipalmitoyl)^14,31^ through different ways like hydration of thin lipid film followed by agitation, sonication, reverse-phase evaporation, and extrusion. Compared with conventional systems, liposomes have a greater therapeutic efficacy (especially for drugs like anticarcinogenics, antifungals, and antibiotics), slower drug release, better patient compliance, less toxicity, and higher biocompatibility.^14,32^

 Skin penetration and stability of liposomes are lower than other nanocarriers like transferosomes and ethosomes.^33^ Having cholesterol in their structure, liposomes have a rigid nature and these nanocarriers are unable to reach deeper layers and release drug in the epidermis.^34^ Although liposomes can be prepared via several methods on a laboratory scale, only a few methods are industrially applicable, the most common of which is ethanol injection followed by extrusion. Extrusion is highly important as the particle size should be strictly controlled. The quality control includes several stages such as pH control at critical steps, filter integrity test, particle size and zeta potential measurements.^35^ The surface is sometimes modified and coated with hydrophilic polymers, like polyethylene glycol (PEG), to prolong the liposomes blood circulation half-lives. Incorporating polymers into the lipid bilayer increases the liposomes’ hydrophilicity and stability in an aqueous environment. Functionalizing the liposomes with targeting ligands can make them more usable in drug delivery for applications like cancer therapy.^36^

 Vemurafenib-resistant melanoma can be treated by protein kinase C inhibitor anchored BRD4 PROTAC PEGylated nanoliposomes. They are formed by using palmitoyl-DL-carnitine chloride (PC) as protein kinase C inhibitor, 1,2-dioleoyl-*sn*-glycero-3 phosphocholine, chloroform, and modified hydration method. In-vitro studies assessed the cytotoxicity, release, stability, angiogenesis (using human umbilical vein endothelial cells), and migration. A novel chemotherapeutic combination was obtained to treat vemurafenib-resistant melanoma, with the PC enhancing the anti-angiogenesis and anti-vasculogenic effect as well as nanoliposomes stability while reducing the size.^37^

 Different formulations of transdermal peptide-modified vemurafenib-loaded liposomes (Vem-TD-Lip) and their inhibitory effects on melanoma cells were studied and examined regarding various features such as intracellular uptake, selective inhibition, penetration, size, and zeta potential. The nanocarriers were found to be regularly cubic, biocompatible, highly stable, capable of selective inhibition of A375 cells, and deliverable via the skin, besides having a low cellular toxicity. Transdermal modification on the liposomes also improved the penetration of vemurafenib. Safety studies showed that compared with oral and intravenous administration of Vem-TD-Lip, local administration caused significantly less damage to the liver, kidney, and lungs. Furthermore, the tumor size, relative volume, and weight after two weeks of administration of Vem-TD-Lip were significantly lower than that in the phosphate-buffered saline.^21^

 Dual receptor-recognizing liposomes with paclitaxel (PTX) and hydroxychloroquine contents were evaluated in treating primary and metastatic melanoma via autophagy. The relative rates of wound healing were 4.82% ± 3.06% and 12.59% ± 4.15% after 24 and 48 hours, respectively. The liposomes increased the drug accumulation in tumor cells, inhibited migration, and reduced lung metastasis *in vivo*. The combination of PTX and hydroxychloroquine was reported as a promising treatment for both primary and metastatic melanoma.^22^

 Gowda et al^30^ assessed the nanoliposomal delivery of A2 inhibitor arachidonyl trimethyl ketone in melanoma treatment by using normal human fibroblast cell lines FF2441, BRAF melanoma cell lines, and random human melanoma specimens. The arachidonyl trimethyl ketone was encapsulated within nanoliposomes via sonication followed by extrusion. Drug stability, entrapment efficiency, *in vitro* drug-release kinetic, cell viability, cellular proliferation and apoptosis, western blot analysis, and tumorigenicity were assessed. Nano arachidonyl trimethyl ketone at 30 and 40 mg/kg significantly reduced the tumor volume by 58% and 55%. The liposomes were more cellucidal against cancer cells than the normal human cells and about one-half less toxic to the control cells than the melanoma cell lines.

###  Niosomes

**Figure 2:**
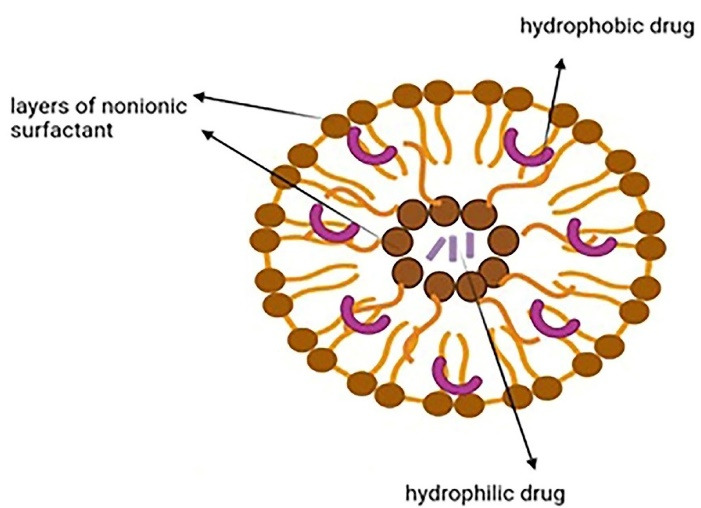
Niosomal structure

 Niosomes are vesicular nanocarriers and synthetic analogs of liposomes that are 100 nm to 2 µm in diameter and have an aqueous core enveloped by several layers of nonionic surfactant. They can carry both hydrophobic and hydrophilic drugs as they are potential carriers for sustained and targeted drug delivery (Figure 2). Niosomes are biodegradable, nonimmunogenic, easily controllable in terms of shape/size/fluidity by altering the compositions, have a large capacity for drug loading, can deliver sensitive drugs through different administration routes (intravenous/transdermal/oral), and show better patient compliance. They are more cost-effective and stable than liposomes. These nanocarriers can deliver several agents like anticarcinogenics, anti-inflammatories, and antimicrobials. Phospholipids and nonionic surfactants enhance penetration as they overcome the barrier of transdermal drug delivery. Different mechanisms are involved such as decreasing the water loss and consequently hydrating the stratum corneum and loosening the cellular structure, or improving the thermodynamic activity gradient of the drug as a driving force for penetration. Some drawbacks are associated with niosomes like physical and chemical instability, aggregation, fusion of the vesicles, and leakage or hydrolysis of the encapsulated drug.^13,38^ Niosomes can be prepared through thin-layer hydration, ether injection, reverse-phase evaporation, lipid injection, and microfluidization.^39^ Surface modification with hydrophilic polymers such as polyethylene glycol can be used for long circulation, while surface decoration with ligands, aptamers, and antibodies is used for target therapy.^40^

 Barani et al^41^ investigated a new formulation of hydrophobin (HFB1)-coated niosomes as carriers of cancer cells by using doxorubicin hydrochloride, Span^®^ 40 (sorbitan monopalmitate), polyethylene glycol, cholesterol, and the thin film hydration method. Characterization of niosomes’ size and morphology via dynamic light scattering, *in vitro* cytotoxic cell assay, and *in vitro* drug release tests were done. The niosomes were found to have almost uniform shape and size, a high and negative zeta potential that yielded a stable formulation, an entrapment efficacy range between 45 and 75% (to be increased by adding vitamin E-acetate), and about 100% doxorubicin release within 4 hours at pH = 7.4, which increased by lowering the pH.

 In an attempt to treat breast cancer, pH-sensitive triaryl-(Z) olefin (TZO as tamoxifen analog)noisome hydrogel was prepared through the handshaking method with components like span 80 and cholesterol (1:1). Crystallinity, entrapment efficacy, stability, and *in vitro* anti-tumor validation were assessed. The results showed that the formulation had low retention in the liver after intra-tumor injection, and the anticarcinogenic activity was more than tamoxifen; hence the formulation was proposed as a useful treatment for breast cancer.^42^

 Mirzaei-Parsa et al^23^ prepared artemether-loaded niosomes via thin film hydration. Cholesterol, tween, and span in different ratios and entrapment efficacy, size, morphology, *in vitro* release, and *in vivo* animal experiments were done. Higher artemether concentration yielded a better inhibitory effect. The tumor volume significantly decreased by day 6 in the noisome-treated group compared with the free drug-treated group. Shortly, the niosomal formulation reduced the tumor volume and increased the necrosis in comparison with the artemether, indicating that being loaded on niosomes could improve the anticarcinogenic effect of artemether.

###  Transferosomes

**Figure 3:**
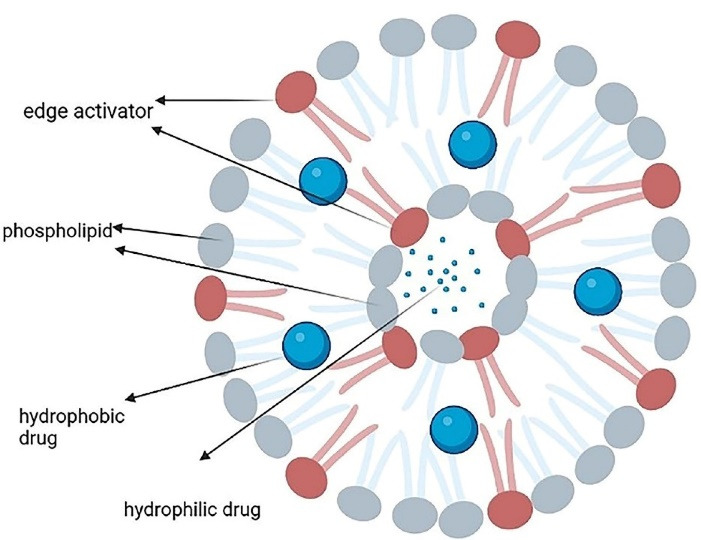
The main structure of a transferosome

 Transferosomes are lipid-based vesicles ( < 300 nm) with highly elastic and formable structures that show stress-response and colloidal stability for up to 3 months. They are comprised of four main parts including phospholipids (like phosphatidylcholine and dipalmitoylphosphatidylcholine), an edge activator (e.g. surfactants or bile salts), < 10% ethanol, and water as a vehicle. The edge activators improve the radius of curvature and increase the membrane deformability, which allows them to squeeze through the channels in the stratum corneum and, therefore, offer a versatile delivery concept. Hydrophilic and hydrophobic drugs can be respectively accommodated in the aqueous core and phospholipid bilayer (Figure 3).^43,44^

 Their preparation methods include rotary film evaporation, reverse-phase evaporation, vortexing sonication, ethanol injection, and freeze-thaw.^45^ Transferosomes can accommodate various drugs such as non-steroidal anti-inflammatory drugs, steroids, and local anesthetics.^46^ Among their strong points are the targeting ability, lower toxicity, prolongation of the drug’s half-lives, and biodegradability. They have high entrapment efficacy, protect the medication against metabolic degradation, and can pass through narrow constrictions; however, they are chemically unstable to oxidative degradation.^47^

 Unfortunately, transferosomal formulations are costly as they are associated with lipid excipients; the most cost-effective option is phosphatidylcholine.^48^ More efforts are required to industrially manufacture transferosomes with the desired composition, stability, loading, and particle size. Although the tolerability of transferosomal formulations has been clinically approved, protocols need to be developed to incorporate this technology in other permeation enhancement techniques such as iontophoresis, electroporation, and microneedles.^43^

 In a study, green synthesized honokiol transfersomes were prepared via the modified scalable heating method and tested for the mean particle size, entrapment efficacy, transmission electron microscopy, *in vitro* drug release, intracellular uptake of fluorescein-loaded transferosomes, cytotoxicity, and some biomarker analyses. The transferosomes showed the potential to alleviate the immunosuppressive characteristics of B16F10 melanoma *in vitro*. They also reduced the expression of the stem-like cell markers CD133, TGFβ, and CD47.^49^

 Paclitaxel-encapsulated cell-penetrating-peptide-modified transfersomes (PTX-CTs) hydrogel for topical melanoma treatment was prepared by using the thin-film dispersion method. Transmission electron microscopy showed that PTX was encapsulated in the lipid bilayer. Evaluating the permeation of the CTs into the mice’s skin via the Franz diffusion cell system showed that the penetration of coumarin 6 fluorescent dye within 12 hours was about 11.4% in the Coumarin 6-loaded transferosomes versus 4.8% in coumarin 6-loaded liposome. *In vitro* comparisons of different PTX formulations revealed that PTX-CTs had higher tumor penetration and cytotoxicity in the B16F10 cells.^24^

###  Ethosomes

**Figure 4:**
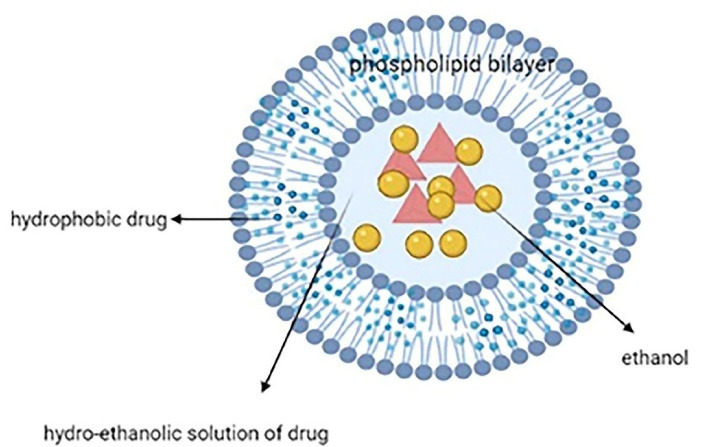
Structure of an ethosomal nanosystem with high ethanol concentration

 Ethosome, another vesicular lipid carrier, is mainly made of a phospholipid bilayer, high ethanol concentration (20%-40%), and an aqueous inner core that entraps the drug (Figure 4). It is much smaller and more flexible than liposomes and a better solution for hydrophobic drugs. Their tiny size (30 nm to several microns) allows them to pass through the skin. They are distinguished from other lipid carriers by their high concentration of ethanol. Other ingredients can be used such as cholesterol (as membrane stabilizer), permeation enhancer, dyes, and gel formers like carbopol and sodium alginate to produce vesicular gels to prolong the residence time.^31,50^ Ethanol can interact with the polar group region of the lipids and causes increased membrane fluidity which calls the ethanol effect. Besides, lipid penetration and permeation occur due to the fusion of the ethosomes with skin lipids, called the ethosome effect. Because of the two effects, ethosomes show improved skin penetration.^51^

 Thanks to some characteristics like bilayer fluidity and lack of side effects, the unique ethosomes system can deliver variable medications like antivirals, anti-inflammatories, and analgesics into deep layers of skin^52,53^; however, dermatitis is probable due to the penetration enhancers like alcohol.^54^ Moreover, their structure improves the entrapment of both hydrophilic and lipophilic drugs. Ethosomes can be simply prepared without special equipment.^55^

 Lin et al^56^ co-loaded the ethosomes with berberine chloride and evodiamine via single-step injection for melanoma treatment. The viability of B16 melanoma cells was 6.25% by using the highest concentration of both drugs (18.6 μg/mL of berberine and 2.25 μg/mL of evodiamine). The findings approved the anti-melanoma effects of the co-loaded ethosomes on B16 cells in tissue culture and are suitable for the treatment of melanoma in an animal model.

 Ma et al^57^ evaluated the polyethyleneimine and sodium cholate-modified ethosomes complex as multidrug carriers (with cytotoxic drugs like doxorubicin and curcumin) for the transdermal treatment of melanoma by making different formulations with materials like fluorescein rescein isothiocyanate, phosphatidylcholine, and rhodamine B.^18^ The thin layer evaporation technique was used and cellular uptake, *in vitro* transdermal performance, and *in vitro* antitumor test were done. The carrier-wrapped drugs had a higher cellular uptake than the nude drug. The anti-tumor activity was higher in the formulations with both doxorubicin and curcumin, with the optimal ratio of 7:3 for polyethylenimine and sodium cholate.

 Yu et al^25^ prepared the transdermal mitoxantrone ethosomal gel by using hydroxypropyl methylcellulose, phospholipid, mitoxantrone, ethanol, and water. The mitoxantrone-loaded ethosomes were made via the thin film dispersion method. Then, characterization of the ethosomes, entrapment efficiency, *in vitro* permeation, cell cytotoxicity on B16 melanoma cells (on an electrical cell-substrate impedance sensing system with a modified chip), and flow cytometric investigation were assessed. *In vitro* permeability across the rat skin, cytotoxicity, and anti-melanoma effect in the mitoxantrone ethosome gel were higher than those of the mitoxantrone solution. The tumor inhibitory rate of the gel was 68.44% and the cell uptake of the mitoxantrone from the ethosomal gel was high. Having no severe side effect, the noninvasive mitoxantrone ethosomal gel was proposed as a perfect drug delivery system for melanoma treatment.

###  Transethosomes

**Figure 5:**
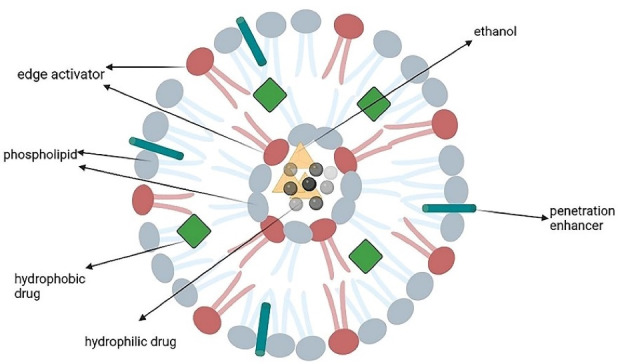
Transethosomes (the combination of ethosomes and transferosomes)

 The novel system of transethosomes (first introduced in 2012) is the combination of ethosomes (lipid and ethanol) and transferosomes (lipid and edge activator) with a combination of the strong points of both systems. They are mainly made of phospholipids, high-concentration ethanol (10%-50%), edge activators (like surfactants), and penetration enhancers like propylene glycol. They are better than their constituents per se in transdermal drug delivery because of their small particle size, high entrapment efficiency, *in vitro* release, and penetration.^58,59^ These nanocarriers have irregular spherical shapes and high vesicle elasticity (Figure 5).^60^ Transethosomes can pass through stratum corneum by adhering to lipid lamella after interacting with the disturbed layer. They show improved flexibility and fluidity due to the ethanol and edge activator and their elastic nature allows them pass through the intercellular pathway.^61^ Nonetheless, dermatitis, allergic reaction, and skin irritation are among the transethosomes disadvantages.^61^

 Abdulbaqi et al^62^ delivered colchicine transdermally through three different formulations of transethosomal gels made with Phospholipon 90G^®^ (PL90G), absolute ethanol, Tween 20^®^, sodium stearate, and triethanolamine via the cold method. The final gel formulation was formed by incorporating colchicine-loaded transethosomes in a pre-prepared gel with a ratio of 4:1 by using a stirrer and then manual mixing. The rheological behavior, ex-vivo skin permeation, and stability were evaluated and transmission electron microscopy was done. The transethosomes had irregular spherical shapes and high entrapment efficacy. The final gel formulations also showed satisfactory stability in refrigerated condition, non-Newtonian plastic flow with no thixotropy, and high skin permeation rate compared with the non-transethosomal gel.

 Song et al^63^ loaded voriconazole on conventional liposomes, deformable liposomes, ethosomes, and transethosomes through the thin film hydration methodand by using materials likeLipoid S100 (phosphatidylcholine soybean lecithin), cholesterol, and taurocholic acid sodium salt (sodium taurocholate). The transethosomes exhibited the highest elasticity (conventional liposomes showed the least), permeation rate (11.5 ± 1 g/cm^2^/h), and skin deposition among all formulations.

###  Cubosomes

**Figure 6:**
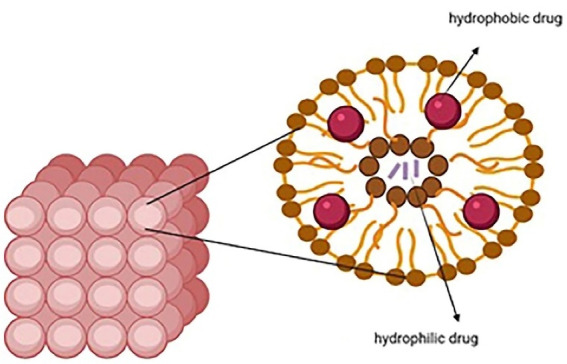
Cubosomes as honeycombed structured nanoparticles

 Cubosomes are honeycomb-structured nanoparticles with internal aqueous channels and a large interfacial area. They are formed by hydrating a surfactant and the subsequent formation of the cubic phase and dispersing a solid-like phase into smaller particles. They are comprised of amphiphilic lipids (usually glyceryl monooleate and phytantriol) that are capable of self-assembly in water and form cubosomes and stabilizer-like pluronics, especially F127 (Poloxamer 407), which is a poly (ethylene oxide)-poly (propylene oxide)-poly (ethylene oxide) triblock copolymer (Figure 6). This nanocarrier can be simply prepared and used in targeting and controlled-releasing bioactive agents. It can load both hydrophilic and lipophilic drugs and be administered via different routes like oral, intravenous, or topical drug delivery. Major advantages of cubosomes are high capacity for drug loading (due to the cubic crystalline structure), biodegradable lipids, and thermodynamic stability; nevertheless, they have drawbacks like low entrapment efficacy for water-soluble drugs.^64-66^ Moreover, pharmaceutical drug delivery has been made possible by the cubic liquid crystalline phases because of the cubosomes cubic nanostructure with hydrophobic and hydrophilic loading capabilities.^67^ Cubosomes can form a thin surface film consisting of a liquid crystal matrix on the mucosal surface in topical drug delivery where nanostructure can achieve an optimal delivery profile and temporary protection of a sensitive skin.^65^

 Cubosomes can be made via either top-down or bottom-up techniques,^68^ the latter is more suitable for large-scale production and is superior to the top-down approach as it requires less energy input and allows working with thermo-sensitive materials.^69^ These nanostructures can be modified by adding biotin or folate, antibody fragment coupling to PEGylated lipids, protein labeling, and coating with poly-ε-lysine.^70^

 By using the top-down method, Cytryniak et al^26^ prepared cubosomes in differentformulationssuch asDOTAGA-oleylamine conjugate **(**DOTAGA-OA), doxorubicin DOTAGA-OA, DOTAGA-OA-177Lu (177Lu = lutetium 177 as radionuclide), and doxorubicin DOTAGA-OA-177Lu. The stability was assessed via dynamic light scattering and found to be acceptable in the doxorubicin DOTAGA-OA-177Lu cubosomes in phosphate-buffered saline. Toxicity was evaluated via the MTS assay and showed that the cubosomes were non-toxic to Hela cells up to 54 mg/mL concentration, with the doxorubicin DOTAGA-OA-177Lu being the most toxic of all cubosomes (36.5 % of the viable cells). That study revealed that the combination of cytotoxic drugs such as doxorubicin and B-emitting radionuclides as cubosomes can improve cytotoxicity. Moreover, with the multifunctional cubosomes, the cytotoxic drugs can be used in smaller doses, and thereby their side effects would be prevented.

 Bazylińska et al^71^ prepared polymer-free cubosomes for simultaneous bioimaging and photodynamic action of photosensitizers in malignant melanoma cells by using monoolein as the building block, phospholipid as the stabilizer, and propylene glycol as the hydrotrope. Morphological and topological features of cubosomes loaded with Chlorin e6 or meso-Tetraphenylporphyrin-Mn (III) chloride (photosensitizing dyes) were examined through dynamic light scattering and transmission electron microscopy. The Ce6-loaded cubosomes reduced the MeWo and Me45 cell viability by 90%. Toxicity was low in both formulations.

 Saber et al^72^ loaded the cisplatin and a combination of cisplatin-metformin on cubosomes through emulsification and targeted the colorectal cancer cell metabolism. Cell culture (human CRC cell lines and HCT116), cytotoxicity assay (using Sulforhodamine B = SRB assay), glucose/ATP/lactase level examination (cells washed in phosphate-buffered saline), lactate dehydrogenase activity, nicotinamide adenine dinucleotide phosphate (NADPH) oxidase activity, AMPK/total MTOR assessment were done. The half-maximal inhibitory concentration (IC_50_) was 15 μM in cisplatin, 9.6 μM in cisplatin-loaded cubosomes, and 7 μM in cisplatin-metformin-loaded cubosomes. The drug uptake was 1.6 greater in the cubosomal formulation of cisplatin. The LDH activity increased more in the cisplatin-metformin-loaded formulation (1.6 times) than that in the cisplatin-loaded cubosomes (1.35 times). The NADPH increased by 3.4 times and the p-AMPK increase was the highest in cisplatin-metformin-loaded cubosomes (7.5 times more).

###  Dendrimers

**Figure 7:**
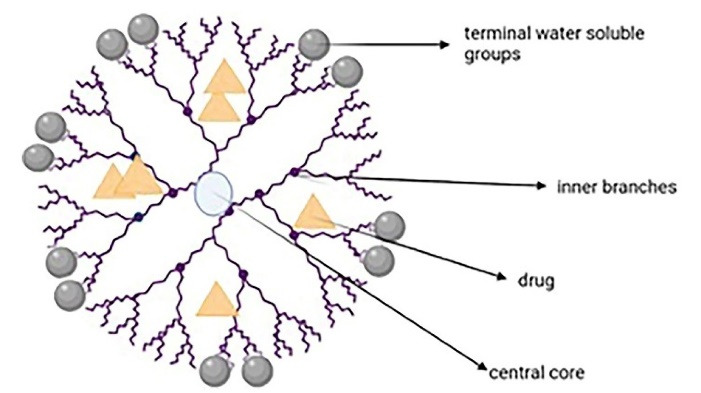
The main parts of a dendrimer

 Dendrimer-based nanocarriers are about to revolve the oncological diagnoses and treatments.^73^ Dendrimers are hyperbranched and tree-like nanocarriers with spherical, globular three-dimensional shapes and high molecular uniformity. Their main three parts are the central core, inner branches, and the terminal surface water-soluble groups (responsible for the perfect water solubility). The inner core encapsulates the drugs, while the surface is suitable for targeting and imaging the ligands (Figure 7). These unique properties have made the dendrimer a potential nanocarrier for diagnosis and drug/gene delivery.^74-76^ Researchers suggest that dendrimers interact with phospholipids via the skin which leads to forming defects or nanoscale holes on cell membrane and also report minimal skin irritation and improved deposition and permeation of many drugs.^77^

 Among the advantages of dendrimers are the surface terminals for conjugating drugs/molecular sensors/biopolymers, monodispersity, and internal space for loading hydrophobic drugs in high capacity. They can be used for passive and active targeting and delivering anticarcinogenic drugs.The amino-terminated dendrimers like poly(amidoamine) (PAMAM) andpolypropyleneimine dendrimers have greater cytotoxicity and intrinsic antiproliferative effect than those with an anionicsurface (like carboxyl, hydroxyl, and acetamido groups). High-density charged groups on the surface of dendrimers such as tertiary amines promote them to perfect vehicles for DNA and siRNA.^74-76^ PAMAM dendrimers have some shortcomings such as poor biocompatibility, high toxicity, and rapid blood clearance due to positively-charged surfaces at biophysiological pH.^8^ Chemical modification of the surface charge is a common approach to control the cellular interactions and biodistributions of dendrimers. Modification of PAMAM dendrimers with neutral or negatively-charged groups has been reported to significantly decrease their toxicity.^79^

 Bhatt et al^80^ prepared different formulations consisting of G4-TOS-PEG-PTX (by conjugating α-Tocopheryl Succinate (α-TOS) and polyethylene glycol on a generation 4 PAMAM dendrimer (G4 PAMAM) and loaded them with PTX), G4-PEG-PTX, G4-TOS-PEG, and other formulations. Entrapment efficacy, *in vitro* release study, hemolytic toxicity, cellular uptake, cytotoxicity, apoptosis, *in vitro* therapeutic efficacy (using female 288 C57BL/6 mice of 6−8 weeks [weight = 18-22 g]), and tumor inhibition were examined. The PTX release from the PTX solution within 8 h (≈99%) was faster than that from the G4-PEG-PTX and G4-TOS-PEG-PTX formulations, showing the controlled release of PTX over 48 h. The G4-TOS-PEG-PTX showed the highest cytotoxicity (viability = 37.21%), and higher apoptosis (27.3%) than the G4-PEG-PTX formulation (40.7%).

 Tassano et al^27^ evaluated the chromosomal aberrations induced by rhenium-188 labeling PAMAM G4 dendrimer (188Re-dendrimer). Biodistribution studies, cell culture, and chromosomal aberration assays (melanoma cells from Mus musculus skin B16F1) were done. The biodistribution assay exhibited high liver uptake (30%) and renal clearance in melanoma-bearing mice. The aberrant metaphase was 29.5% in cells treated with 15 μCi (0.555 MBq) of the 188Re-dendrimer for 24 hours and 7% in the control cells. Accordingly, the G4 PAMAM dendrimers were reported radiolabeled with 188Re through the bifunctional HYNI chelating agent and capable of inducing chromosomal aberration in malignant cells.

 Zhu et al^81^ studied different PEGylated PAMAM conjugated dendrimers (PPSD and PPCD conjugates by using acid-sensitive cis-aconityl linkage and acid-insensitive succinic linkage between doxorubicin and polymeric carriers) with different PEGylation degree for tumor-selective targeting of doxorubicin. The cytotoxicity assay exhibited that PAMAM dendrimers were cytotoxic to B16 cells with IC_50_ = 1.95 mM; PEGylation reduced the cytotoxicity and increased IC_50_. The cellular uptake of doxorubicin in PPSD conjugates was remarkably higher than that in PPCD conjugates. All of the formulations could alter the pharmacokinetic parameters in comparison with doxorubicin solution like increasing AUC and all of them were effective in the prevention of tumor growth.

###  Cyclodextrins

**Figure 8:**
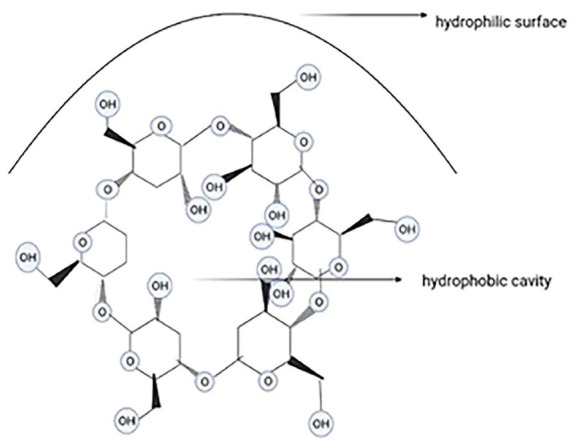
α-cyclodextrin with 6 glucose units

 Cyclodextrins are cyclic and cone-shaped oligosaccharides (with a hydrophobic cavity due to the CH_2_ groups and a hydrophilic surface due to the secondary hydroxyl functional groups)with 6, 7, or 8 glucoseunits, known as α, β, and cyclodextrin, respectively (Figure 8). Cyclodextrins have been remarkably used in gene therapy, cell therapy, immunotherapy, and chemotherapy. These nanocarriers improve the water solubility and stability of drugs and decrease their toxicity to human bodies. Given the overexpression of some ligands like folate receptors on tumor cells, modified cyclodextrins (because of their hydroxyl groups) can be used for targeted therapy. Preparation and storage of cyclodextrins are restricted because of their instability, sublimation, and low aqueous solubility.^82,83^ Solvent evaporation, slurry method, and dry mixture are some methods of preparing cyclodextrins.^84^ Cyclodextrins improve absorption by solubilizing the drug at the absorption cite and the interactions with the free lipids in stratum corneum.^85^ Despite the numerous cyclodextrin derivatives, only a few can be synthesized in industrial-scale, because the complicated multi-step reactions and purification of the products by chromatography restrict them to laboratory scales is possible. The most important βCD-derivatives are the methylated βCDs which are heterogeneous, amorphous, and highly aqueous-soluble.^86^ The delivery capability of the CD molecules can be enhanced by scaffolding them with a polymer graft with cationic surface charges. βCD not only reduces the toxicity of the grafted polymer but also enhances membrane absorption and molecular stabilization. Polyethylenimine is the gold standard of gene transfection and CD-modified polyethyleneimine derivatives have lower toxicity.^87^

 Ferraz et al^28^ prepared Harman (HAR) βCD in a 1:1 molar ratio and characterized via FTIR, NMR, and SEM. Entrapment efficiency, cell viability of normal cells assay (using MTT after 24 hours), cell migration assay, apoptosis analysis (using caspase-3 activity assay), and sensitization of A2058 cell to chemotherapy (using MTT) were done. In a nutshell, those findings revealed that βCD-HAR would promote the toxicity more than HAR per se with an IC_50_ = 23.36 μM. HAR and βCD-HAR decreased the migration rate by 32.23% and 46.37%, respectively. Caspase-3 activity was higher in the cells treated to βCD-HAR compared with the HAR-treated cells. The ability of HAR and βCD-HAR to sensitize the A2058 cells was determined by combining them with dacarbazine, vemurafenib, and fluorouracil; they were found to increase the cytotoxic effects of anticarcinogenic drugs.

 Sun et al^88^ prepared curcumin-hydroxypropyl-βcyclodextrin (Cur/HP-βCD) for melanoma treatment and the solubility was measured. Then, in-situ hydrogels were made with poloxamer 188 and 407. Photochemical stability, curcumin release, permeation, cytotoxicity, and flow cytometry were evaluated. These cyclodextrins increased the stability (from 0.15 to 3.09 mg/mL) and solubility (from 0.02 to 0.15 mg/mL). The HP-βCD could also protect the curcumin from degradation. The IC_50_ was 702.27 µM in curcumin and 281.88 µM in CUR/HP-βCD group, confirming the inhibitory effect of curcumin complexes. Briefly, this study demonstrated that cyclodextrins improved solubility, stability, curcumin release, transdermal permeation, and cell toxicity.

 Pizzimenti et al^89^ prepared hydroxynonenal (HNE) with a derivative of βCD [βCD-poly(4-acryloylmorpholine) conjugate [PACM-βCD] and assessed for *in vitro* release, stability (over 12 months), cell culture (A375), cell viability, and cellular uptake (via confocal laser scanning microscopy). The cyclodextrin reduced the number of viable cells in comparison with free HNE. Ten µg of the formulation induced apoptosis in HL-60, PC3, and A357 cells in 24 h compared with the free HNE-treated cells that showed very low apoptosis.

 Michel et al^90^ prepared different formulations of Gemini surfactant-conjugated cyclodextrins and incorporated with poorly-soluble curcumin. The size and zeta potential measurements, cell toxicity assays, Caspase-Glo^®^ 3/7 assays, and mass spectroscopy were done.^73^ The IC_50_ values for the treated cells were about 0.80 µM for the drug/ cyclodextrins and 0.86 µM for the drug/ cyclodextrin gemini formulations. For the apoptotic assays, the Caspase 3 and 7 as apoptosis markers were evaluated. Shortly, this study showed that this formulation was effective in melanoma cell inhibition with IC_50_ lower than the melphalan and a low toxicity on human epidermal keratinocytes.

###  Solid lipid nanoparticles

**Figure 9:**
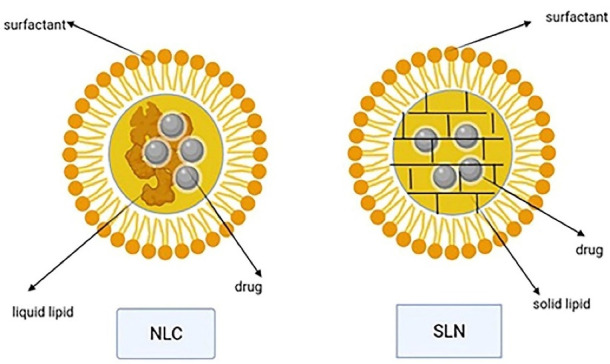
NLC and SLN structures with liquid and solid lipid

 Transdermal use of the solid lipid nanoparticles and nanostructured lipid carriers has yielded promising results.^91^ The solid lipid nanoparticles (with a size between 40 and 1000 nm) is a substitution for polymeric nanoparticles like liposomes. These systems mainly consist of 0.1-30% w/w solid lipids dispersed in an aqueous medium and a surfactant (0.5-5% w/w) as a stabilizer. The nanostructured lipid carriers with a 40-50% liquid lipid content were developed as nanoparticles with a higher loading capacity and a lower water content of the particle suspension compared to solid lipid nanoparticles (its water content was 70-99.9%). They are made of solid and liquid lipids mixed in a ratio of 70:30 to 99.9. The lipids used to prepare these nanoparticles are triglycerides, fatty acids, waxes, and glycerides (Figure 9).^92,93^

 Both solid lipid nanoparticles and nanostructured lipid carries are perfect transdermal carriers due to features like biodegradable lipids, low toxicity, high drug penetration, and occlusive properties that increase skin hydration. Lipid nanoparticles can also enhance the stability of agents that are sensitive to oxidation, light, and hydrolysis. Different preparation techniques are available, the common step of all of which is the formation of oil in water precursor followed by solidification of the dispersed solid phase.^92,93^ Occlusion effect after the lipid film formation on the skin has been reported for lipid nanoparticles. It reduces transepidermal water loss and consequently causes skin hydration, which in turns decreases corneocytes packing and increases the size of corneocytes gaps and facilitates the drug penetration to deeper skin layers.^94^ Particle growth, unpredictable gelation tendency, and burst release are some disadvantages of solid lipid nanoparticles.^95^ Different studies demonstrated that surface modification of solid lipid nanoparticles with ligands for receptors overexpressed on the surface of cancer cells can improve the uptake of this nanocolloidal drug carrier in the tumor site.^96^ By using the high-pressure homogenization method, large-scale production can be obtained in a cost-effective and relatively simple way.^97^

 In Banerjee and colleagues’ study,^29^ solid lipid nanoparticles modified with Tyr-3-octreotide (PSM) formulation of PTX in a murine melanoma model were studied and cell apoptosis, starch assay, biodistribution, antimelanoma efficacy, cytokine measurement (ELISA), and therapeutic efficacy were studied. The necrosis and apoptosis rate was higher in the PSM group than that in the DTIC group. Both PSM and DTIC treatments demonstrated cellular shrinkage and rounding. The effects of PSM and DTIC on cell migration were examined by starch assay and the wound healing was prolonged in the PSM-treated group. The PSM elevated levels of IFN-γ, IL-2, IL-12, and TNF-α and showed the least lung metastasis in mouse models.

 Goto et al^98^ prepared aluminum chloride phthalocyanine-loaded (CIAIPC as a photosensitizer) solid lipid nanoparticles for photodynamic inactivation of melanoma cells via direct emulsification method and two surfactants, naming sorbitan isostearate and polyoxyethylene-40 hydrogenated castor oil. Encapsulation efficacy, biocompatibility, stability, photodynamic therapy efficacy, and MTT assay for cell viability were examined. A stability test using the size and zeta potential at 4 degrees showed that CIAIPC SLN was physically and chemically stable for 24 months. It also had a dose-dependent phototoxic effect on melanoma cells.

###  Inorganic nanocarriers

**Figure 10:**
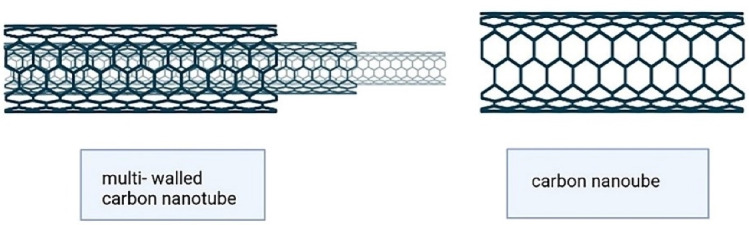
Carbon nanotubes (single and multi-walled structure)

 These inorganic nanocarriers are currently considered for theranostic processes because of their large surface area, high capacity for drug loading, biocompatibility, and low side effects. They encompass quantum dots, gold nanoparticles, silver nanoparticles, graphene oxide nanosheets, iron oxide nanoparticles, mesoporous silica nanoparticles, and carbon tubes, to name a few. For instance, carbon nanotubes (CNTs) are ordered tubular structures that have a nano-sized diameter. These nanocarriers are made by a single or more layers of carbon atoms as coiled sheets of graphene (Figure 10). According to the acidic and lipophilic nature of stratum corneum, inorganic nanoparticles with positive charge, high surface lipophilicity, and small particle size could penetrate this layer of skin and reach the deeper layers.^99^

 Their surfaces or edges can be functionalized by different groups, which improves the solubility, absorption, distribution, and pharmacodynamic properties. The CNTs can be used as the biocompatible vehicle of different anticarcinogenic agents for transdermal drug delivery. Considering all of these aspects, CNTs are a proper candidate for melanoma treatment.^100,101^ Because of their excellent mechanical, thermal, electrical, and optical properties, CNTs have been used for killing malignant cells in previous studies.^20,102-104^ Carbon nanotubes are not soluble in aqueous media; thus, functionalization is essential for their biological and biomedical applications.^105^ Table 4 summarizes the advantages, disadvantages, and specific characterization of all studied nanotechnology-based drug delivery systems.

**Table 4:** Advantages, disadvantages, and specific characterization of nanotechnology-based drug delivery systems for melanoma transdermal therapy

**Nanotechnology-based drug delivery systems**	**Advantages**	**Disadvantages**	**Specific characterization**
Liposome	Greater therapeutic efficacy, slow drug-release, better patient compliance than the conventional systems, less toxic, and biocompatible.	Less skin penetration and they are less stable than other nanocarriers like transferosomes and ethosomes.	
Niosomes	Easily-controlled shape/size/fluidity by altering the compositions, a large amount of drug loading, delivery of sensitive drugs, administration via different routes, and better patient compliance. Lower cost and more stability than liposomes.	Physical and chemical instability, aggregation, fusion of vesicles, and leaking or hydrolysis of the encapsulated drug.	Phospholipids and nonionic surfactant act as penetration enhancers that can overcome the barrier of transdermal drug delivery.
Transferosome	Ultraformable, elastic, and stress-responsive vesicles in comparison with liposomes and niosomes. The greatest colloidal stability, biodegradability, high entrapment efficacy, protection of the drug against metabolic degradation.	Expensive cost and chemically-unstable to oxidative degradation.	Edge activators improve the radius of curvature and increase the deformability in the membrane and this lets them squeeze through channels in SC and offers a versatile delivery concept.
Ethosome	much smaller and more flexible than liposomes	Dermatitis can happen in some patients due to penetration enhancers like alcohol	Ethosomes can pass through the skin because of their small size.
Transethosome	Transethosomes are better than ethosomes or transferosomes to transdermally deliver drugs because of their small particle size, high entrapment efficacy, high *in vitro* release, and penetration.	Dermatitis, allergic reaction, and skin irritation.	
Cubosome	High drug loading (because of the cubic crystalline structure), biodegradable lipids, and thermodynamically stable.	Low entrapment efficacy for water-soluble drugs	Honeycombed structured
Dendrimer	Having surface terminals for conjugating drugs/molecular sensors/biopolymers, monodispersity, and internal space for loading hydrophobic drugs in high capacity.	Poor biocompatibility, high toxicity, and rapid blood clearance, due to their positively charged surfaces at biophysiological pH for PAMAM dendrimers.	Potential nanocarrier for diagnosis and drug/gene delivery. High density charged groups on the surface of dendrimers such as tertiary amines make them perfect vehicles for DNA and siRNA.
Cyclodextrin	They can be used in gene therapy, cell therapy, immunotherapy, and chemotherapy	instability, sublimation, and low water solubility	The hydroxyl groups allow targeted therapy.
Solid lipid nanoparticle	Consisting of biodegradable lipids, low toxicity, can enhance stability of agents that are sensitive to oxidation, light, and hydrolysis	Particle growth Unpredictable gelation tendency Occasional burst release	High drug penetration Occlusive properties that increase the skin hydration.
Carbon nanotube	Improved solubility, absorption, distribution, and pharmacodynamic properties	Not soluble in aqueous media, so the process of functionalization is essential for their biological and biomedical applications.	

## Conclusion

 With respect to the analysis of the reviewed articles, it can be concluded that melanoma treatment can benefit from the advantages of novel nanocarriers like liposomes, niosomes, and transferosomes without the adverse effects of conventional therapies. Further nanotechnology studies can be conducted in-vivo on human or animal models to assess cell toxicity of these agents in treatment of melanoma.

## Acknowledgments

 Appreciations are expressed to Ms. Farzaneh Rasooli for copyediting and improving the English structure of this manuscript.

## Competing Interests

 There is no conflict of interest.

## Ethical Approval

 Not applicable.
